# Serum amyloid A promotes emphysema by triggering the reciprocal activation of neutrophils and ILC3s

**DOI:** 10.1002/ctm2.637

**Published:** 2021-12-19

**Authors:** Jihyun Kim, Jae Woo Shin, Hyun‐Jun Lee, Ji Hyung Kim, Sun Mi Choi, Chang‐Hoon Lee, Hye Ryun Kang, Seok Hee Park, Yoe‐Sik Bae, Doo Hyun Chung, Hye Young Kim

**Affiliations:** ^1^ Laboratory of Mucosal Immunology Department of Biomedical Sciences Seoul National University College of Medicine Seoul South Korea; ^2^ Seoul National University Medical Research Center Institute of Allergy and Clinical Immunology Seoul South Korea; ^3^ Natural Medicine Research Center Korea Research Institute of Bioscience and Biotechnology (KRIBB) Cheongju Chungbuk South Korea; ^4^ College of Life Sciences and Biotechnology Korea University Seoul South Korea; ^5^ Department of Internal Medicine Seoul National University Hospital Seoul South Korea; ^6^ Department of Biological Sciences Sungkyunkwan University Suwon South Korea; ^7^ Samsung Advanced Institute for Health Sciences and Technology Sungkyunkwan University Seoul Korea; ^8^ Department of Pathology Seoul National University College of Medicine Seoul South Korea; ^9^ Laboratory of Immune Regulation Department of Biomedical Sciences Seoul National University College of Medicine Seoul South Korea

Dear Editor,

Chronic obstructive pulmonary disease (COPD) is a group of irreversible lung diseases that include emphysema and chronic bronchiolitis.^1^ Many studies have shown that COPD is due to adaptive immune responses, particularly CD8^+^ and CD4^+^ T cells^2^, ^3^; however, the role(s) that innate immune cells such as innate lymphoid cells (ILCs) and granulocytes play in the pathogenesis of emphysema and COPD is largely unknown. Here, we show that neutrophils, and particularly type 3 innate lymphoid cells (ILC3s), play a key role in the development of COPD with the emphysema phenotype.

To explore the roles of ILCs in COPD pathogenesis, we induced the chronic phase of emphysema by treating lipopolysaccharide (LPS) and porcine pancreatic elastase (PPE) in the Rag1^–/–^ (which lack mature B and T cells) and wildtype (WT) mice for 4 weeks (Figures 1A and S1A). This treatment induced emphysema‐like changes in the lung (Figures 1B and S1B). Since previous studies suggest that emphysema associates with elevated circulating serum amyloid A (SAA) levels,^4^, ^5^ we examined the mRNA expression of SAA and found that *Saa3* was particularly increased in the emphysematous lung but not in the liver (Figure 1C). Induction of SAA was also confirmed by flow cytometry (Figures 1D,E and S1C,D). Moreover, the level of hSAA1, whose amino acid sequence is most similar to that of mouse SAA3,^6^ is higher in the sputum of emphysematous patients than in the sputum of non‐emphysematous COPD patients (Table S1 and Figure 1F). Monocytes and dendritic cells were the main cellular sources of SAA in the emphysema‐induced lungs (Figures 1G,H and S1E,F). Under the same condition, both interferon (IFN)‐γ^+^ and interleukin (IL)‐17A^+^ILCs are significantly increased (Figures 1I,J and S1G,H). The same trend for increasing SAA and ILC3s was observed in the acute model of emphysema (Figure S2A–E). Consistent with this, the frequency of ILC3s in the sputum of emphysematous COPD patients was higher than that of non‐emphysematous patients (Figure 1K).

**FIGURE 1:**
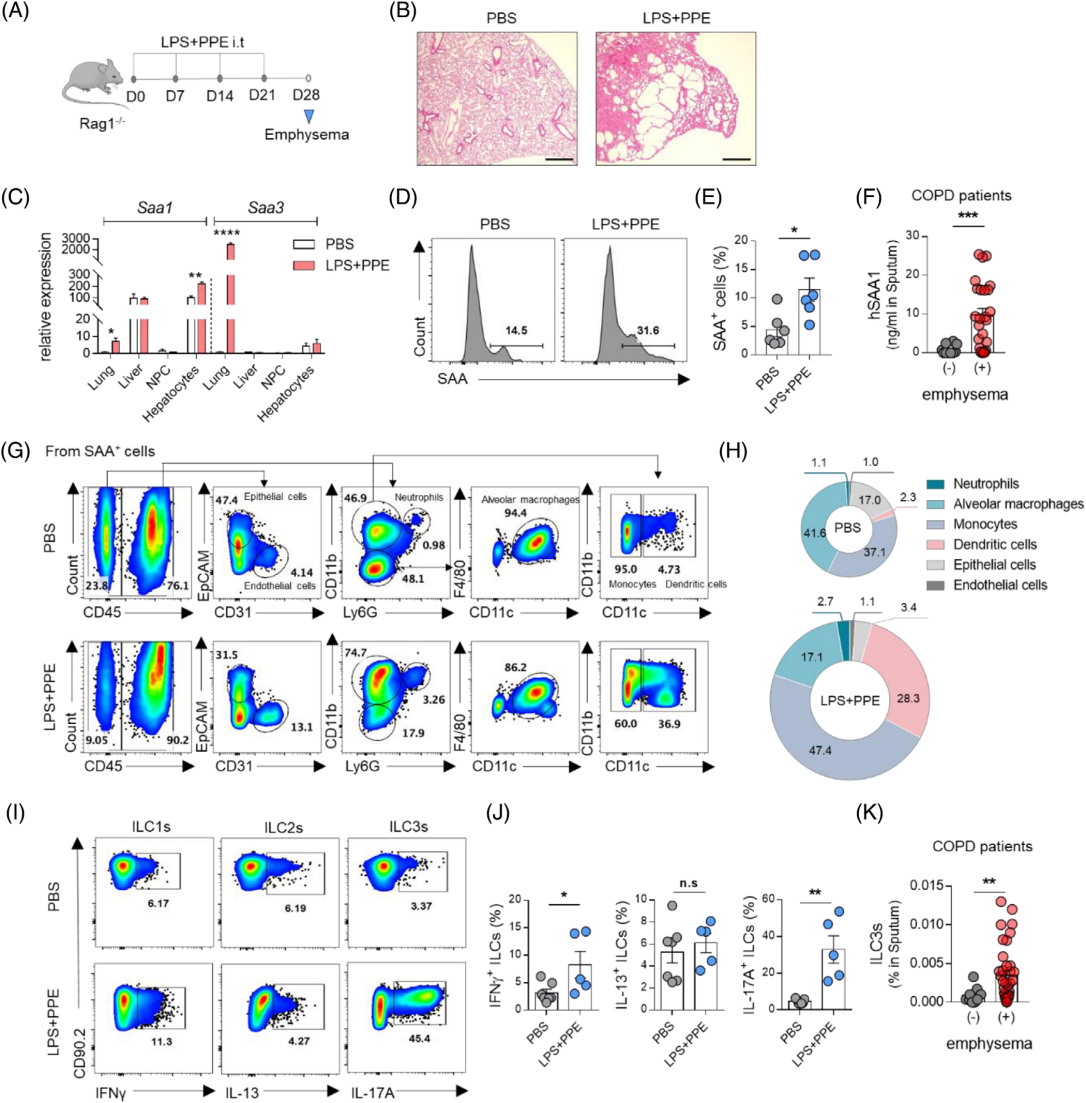
SAA and ILC3s are increased in mouse models of emphysema. (A) Schematic representation of the experimental protocol. To model the chronic phase of emphysema, Rag1^–/–^ mice were treated with lipopolysaccharide (LPS) and porcine pancreatic elastase (PPE) at Days 0, 7, 14 and 21, and then sacrificed 7 days after the final dose. (B) Representative images of lungs from phosphate‐buffered saline (PBS)‐ or LPS/PPE‐treated Rag1^–/–^ mice. Scale bars, 500 μm. (C) The relative expression of *Saa1* and *Saa3* from lungs and livers including non‐parenchymal cells and hepatocytes after induction of emphysema. (D, E) Flow cytometry analysis of SAA^+^ cells in Rag1^–/–^ mice (D) and the frequency of SAA^+^ cells (E). (F) The level of SAA1 in induced sputum of chronic obstructive pulmonary disease (COPD) patients without emphysema (*n* = 9) and COPD patients with emphysema (*n* = 41). (G) The source of SAA in Rag1^–/–^ mice in emphysema model. (H) Pie chart of the percentages of cells secreting SAA in the lungs of Rag1^–/–^ mice. (I) Representative flow cytometry dot plots of IFN‐γ, IL‐13 and IL‐17A production by innate lymphoid cells (ILCs; CD45^+^Lin^–^CD90.2^+^ cells). (J) Quantification of production of cytokines from ILCs. (K) The proportion of ILC3s (CD45^+^Lin^–^CD127^+^ST‐2^–^C‐kit^+^) in the induced sputum of the COPD patients without emphysema (*n* = 9) and COPD patients with emphysema (*n* = 41). n.s.: non‐significant, *p ≤ .05, **p ≤ .01, ***p ≤.001 and ****p ≤ .0001, statistically analysed by unpaired *t*‐test. The data are representative of 2–3 independent experiments and are presented as the mean ± SEM

Next, we asked whether SAA itself could provoke emphysema and increase ILC3s. Even a single dose of rhSAA1 caused acute inflammation and emphysema in the lung (Figure 2A,B). Remarkably, the administration of rhSAA1 specifically increased the actively proliferating IL‐17A^+^ILC3s (Figure 2C–E). In contrast, there were minimal changes in the T_H_ cells (Figure S3A,B). To test whether SAA directly promoted ILC3 expansion, we treated naïve ILCs with rhSAA1 in vitro and found that rhSAA1 did slightly increase the number of IL‐17A^+^cells (Figure 2F,G). Since the increase in ILC3s by in vivo rhSAA1 treatment was much higher (Figure 2D), factors other than SAA may induce the proliferation of ILC3s. To identify those factors, we compared the expression of innate cytokines that can stimulate ILCs and found that only *Il1b* expression was elevated after rhSAA1 administration (Figures 2H and S4A). The increase in IL‐1β by rhSAA1 was also confirmed by flow cytometry (Figure 2I,J), and IL‐1β‐producing cells were mostly neutrophils (Figures 2K,L and S4B). When naïve neutrophils derived from bone marrow were stimulated with rhSAA1 in vitro, the expression of *Il1b* (Figure 2M) and of receptors that are known to recognise SAA, namely, formyl peptide receptor 2 (*Fpr2*), Toll‐like receptor 2 (*Tlr2*) and *Tlr4* ^7^ was increased (Figure S4C). However, this upregulation was not observed in ILCs (Figure S4D). Moreover, the frequency of neutrophils in the sputum was higher in COPD patients with emphysema (Figure 2N). In the sputum, neutrophils and IL‐1β show a positive correlation, and IL‐1β was elevated in emphysematous COPD patients (Figure 2O,P).

**FIGURE 2:**
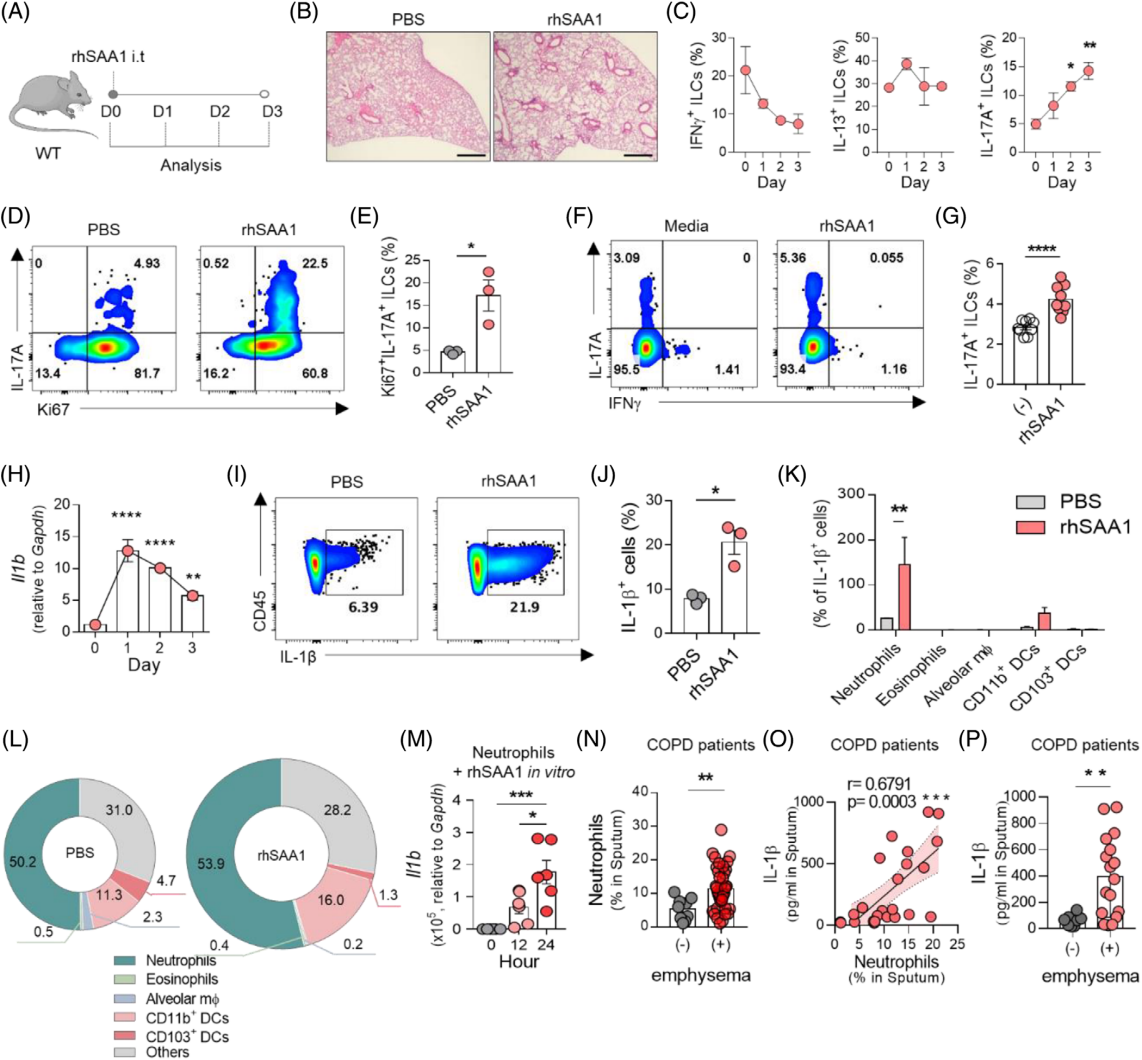
ILC3s are increased in the SAA‐induced emphysema model. (A) Schematic diagram of the SAA‐induced emphysema model. Recombinant human SAA1 (5 μg) was injected intratracheally at Day 0. (B) Microscopic analysis of the lungs of mice with SAA‐induced emphysema on Day 3 by hematoxylin and eosin (H&E) staining. Scale bars, 500 μm. (C) Kinetic analysis of IFNγ, IL‐13 and IL‐17A^+^ILCs after rhSAA1 injection. (D) Ki67 expression by IL‐17A^+^ILCs at Day 3. (E) Comparison of Ki67‐expressing IL‐17A^+^ ILCs in PBS‐ and rhSAA1‐injected mice at Day 3. (F) Flow cytometry of IL‐17A^+^ILCs 3 days after in vitro rhSAA1 treatment. (G) Comparison of IL‐17A‐producing ILCs 3 days after in vitro SAA treatment. (H) Expression of *Il1b* in the lungs of mice with SAA‐induced emphysema. (I,J) Comparison of the frequency of IL‐1β^+^ cells between PBS‐ and SAA‐injected mice; dot plot (I) and quantification (J). (K) The number of IL‐1β^+^ cells in the lungs of mice with SAA‐induced emphysema. (L) Pie chart of IL‐1β secreting immune cells in the lung; cells were gated as Figure S4B. (M) Expression of *Il1b* in bone marrow‐derived neutrophils after SAA treatment in vitro. (N) The proportion of neutrophils (CD45^+^CD68^–^SSC‐A^high^CD16^+^CD24^intermediate^) in the induced sputum of COPD patients without emphysema (*n* = 9) and COPD patients with emphysema (*n* = 41). (O) Correlation between neutrophils and IL‐1β level in sputum. (P) Comparison of IL‐1β level in sputum between non‐emphysematous COPD patients and emphysematous COPD patients. *p ≤ .05, **p ≤.01, *** *p* ≤.001 and ****p ≤ .0001, analysed by unpaired *t*‐test and one‐way ANOVA followed by Bonferroni's post‐test. The data are representative of 2–3 independent experiments and are presented as the mean ± SEM

Based on these results, we hypothesised that neutrophils recognise SAA and secrete the ILC3‐stimulating cytokine IL‐1β, thereby inducing ILC3s to proliferate. To test this, we depleted the neutrophils or blocked IL‐1β and confirmed that these treatments significantly improved the emphysema phenotypes (Figure 3A,B). Moreover, the depletion/blocking of neutrophils or IL‐1β drastically decreased Ki67^+^IL‐17A^+^ILC3s in the lungs (Figure 3C,D). Next, we depleted ILCs to determine their role in emphysema using Rag1^–/–^ mice (Figure 3E). ILC depletion not only reduced IL‐17A‐producing ILC3s but also significantly ameliorated the histological features of emphysema (Figure 3F–H). Notably, ILC depletion did not affect the infiltration of neutrophils into the lung (Figure 3I,J). These results suggest that ILC3s rather than neutrophils are critical for the development of emphysema.

**FIGURE 3:**
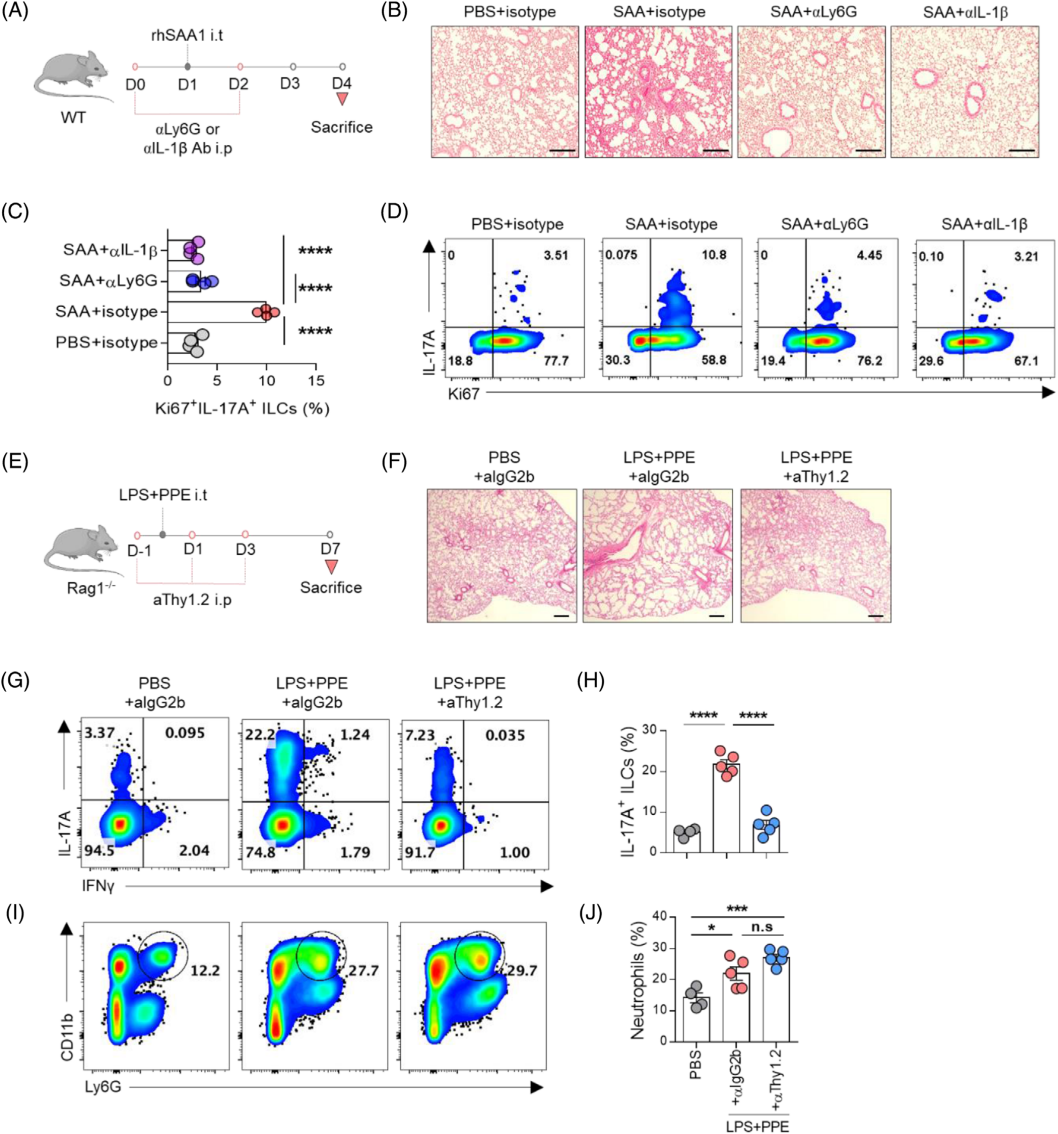
Neutrophil‐derived IL‐1β activates ILC3s in the SAA‐induced emphysema model and IL‐17A^+^ ILCs are essential for the development of emphysema. (A) Schematic diagram of Ly6G blocking and IL‐1β neutralisation in the SAA‐induced emphysema model. (B) H&E staining of the lungs after anti‐Ly6G or anti‐IL‐1β antibody administration in the SAA‐induced emphysema model. Scale bars, 500 μm. (C) Comparison of the frequency of Ki67^+^IL‐17A^+^ ILCs after anti‐Ly6G or anti‐IL‐1β antibody treatment. (D) Ki67^+^IL‐17A^+^ ILCs after anti‐Ly6G or anti‐IL‐1β antibody treatment in the SAA‐induced emphysema model. (E) Schematic diagram of ILC depletion with a Thy1.2 antibody in the acute phase of LPS and PPE‐induced emphysema in Rag1*^–/–^* mice. (F) H&E staining of the lungs after Thy1.2 antibody administration in LPS and PPE‐induced emphysema. Scale bars, 500 μm. (G) Representative dot plots of IL‐17A^+^ ILCs after Thy1.2 antibody administration. (H) Quantitation of IL‐17A^+^ ILCs after Thy1.2 antibody administration. (I) Representative dot plots of neutrophils after Thy1.2 antibody treatment. (J) Quantitation of neutrophils after Thy1.2 antibody administration. n.s.: non‐significant, *p ≤ .05, ***p ≤ .001 and ****p ≤ .0001, by unpaired *t‐*test and one‐way ANOVA followed by Bonferroni's post‐test. The data are representative of 2–3 independent experiments and are presented as the mean ± SEM

To understand how ILC3s induced the emphysema, we first measured the gene expression of matrix metalloproteinases (MMPs)–1, 2, 8, 9 and 12, which play essential roles in the development of emphysema.^8^ Only *Mmp12* expression was significantly increased in the lungs from LPS/PPE‐treated Rag1^–/–^ mice (Figure 4A) and reduced by ILC depletion (Figure 4B). Although alveolar macrophages expressed the highest MMP12 levels, they did not change with emphysema. Instead, neutrophils significantly increased the expression of MMP12 in the emphysema condition (Figure 4C,D). Therefore, we hypothesised that ILC3s induce neutrophils to express MMP12 in the emphysematous lung. To test this, we co‐cultured naïve neutrophils with activated ILC3s and measured the expression of MMP12 (Figure 4E). MMP12 expression in the neutrophils was low but elevated when neutrophils were co‐cultured with activated ILC3s (Figures 4F and S5A‐B). This was also confirmed by flow cytometry, and IL‐17 blockade abrogated the MMP12 expression in the neutrophils (Figure 4G,H). Thus, ILC3s induce emphysema by promoting the MMP12 secretion of neutrophils. Finally, we asked whether this SAA‐neutrophil‐ILC3 axis is also involved in the pathogenesis of the emphysema phenotype in COPD patients. SAA1, neutrophils and ILC3s were increased in the induced sputum from emphysematous COPD patients and were positively correlated with each other (Figure 4I–K). Moreover, lung function was decreased significantly as the lung neutrophils and ILC3s increased (Figure 4L–N). Together, these data suggest that the SAA‐neutrophil‐ILC3 axis is also applicable to COPD patients with emphysema.

**FIGURE 4:**
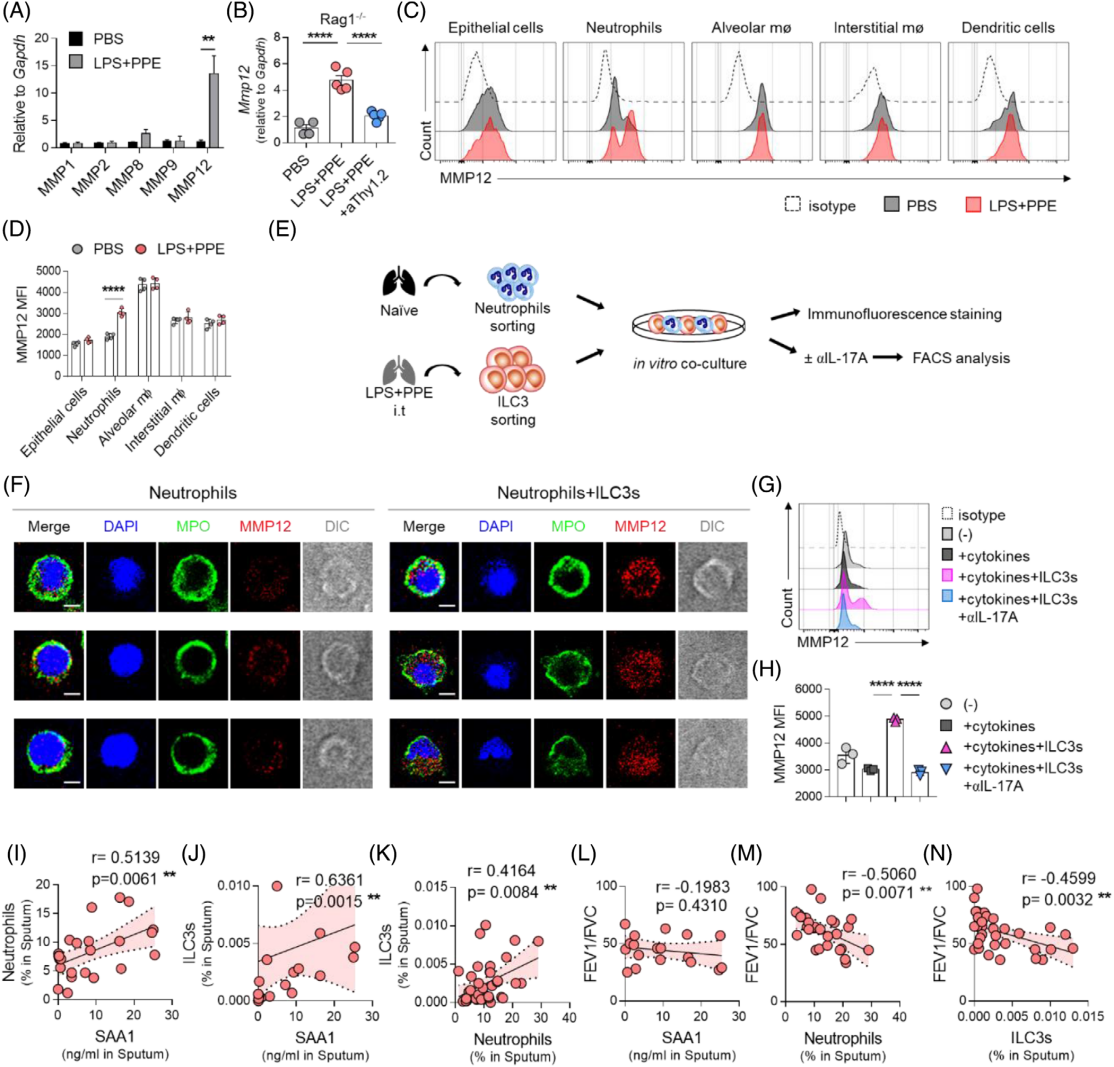
ILC3s induce the expression of matrix metalloproteinase‐12 (MMP12)from neutrophils in emphysema. (A) The relative gene expression of MMPs from lungs of LPS and PPE‐induced emphysema model. (B) The expression of Mmp12 from lungs of Rag1^–/–^ mice after treatment of LPS and PPE with anti‐Thy1.2 blocking antibody (as in Figure 3E). (C) Intracellular staining of MMP12 from epithelial cells (CD45^–^EpCAM^+^), neutrophils (CD45^+^CD11b^+^Ly6G^+^), alveolar macrophages (alveolar mø; CD45^+^CD11b^–^Ly6G^–^SiglecF^+^), interstitial macrophages (interstitial mø; CD45^+^Ly6G^–^SiglecF^–^CD11c^–^F4/80^+^), and dendritic cells (CD45^+^Ly6G^–^SiglecF^–^F4/80^–^CD11c^+^MHCII^+^) in lungs of LPS and PPE‐induced emphysema model. (D) Mean fluorescence intensity (MFI) of MMP12 from each cell population. (E) Schematic diagram of co‐culture of naïve neutrophils from lungs and activated ILC3s from LPS and PPE‐treated lungs. To activate ILC3s, the combination of rmIL‐2, rmIL‐7, rmIL‐1β and rmIL‐23 was added, and to neutralising IL‐17A, anti‐IL‐17A neutralising antibodies were treated. (F) Immunofluorescence staining of neutrophils after co‐cultured with ILC3s. Anti‐myeloperoxidase (MPO) (green), anti‐MMP12 (red) and 4',6‐diamidino‐2‐phenylindole (DAPI) (blue) were stained for analysis. Scale bars, 2 μm. (G,H) Comparison of MMP12 expression from neutrophils after co‐cultured with ILC3s and anti‐IL‐17A neutralising antibodies. Histogram (G) and MFI (H). (I,N) The correlation coefficients were analysed in the induced sputum of the COPD patients with emphysema; Correlation between the level of SAA1 and neutrophils (I), between the SAA1 level and ILC3s (J), between neutrophils and ILC3s (K), between SAA1 level and the FEV_1_/FVC ratio (L), between neutrophils and the FEV_1_/FVC ratio (M), and between ILC3s and the FEV_1_/FVC ratio (N). **p ≤ .01 and ****p ≤ .0001, by unpaired *t*‐test, one‐way ANOVA followed by Bonferroni's post‐test and the Spearman correlation coefficients. The data are representative of 2–3 independent experiments and are presented as the mean ± SEM.

In conclusion, although the role of ILC3s in lung disease has been underestimated due to the rarity, the current study suggested that lung‐resident ILC3s may be critical regulators of emphysema and perhaps also acute exacerbation of chronic obstructive pulmonary diseases. The present study showed that ILC3s and neutrophils reciprocally interact to induce emphysema, but ILC3s may be the key emphysema‐inducing cells: This is shown by the fact that (1) ILC3 depletion improved the emphysema even in the presence of neutrophils, and (2) ILC3 depletion in emphysema‐induced mice eliminated the ability of neutrophils to produce MMP12. Therefore, ILC3‐neutrophils could be applied to COPD patients with emphysema through IL‐17A‐based immunotherapy. To this end, how ILC3s are specifically targeted to modulate local immune responses requires further exploration.

## FUNDING INFORMATION

This study was supported by grants from the Korea Healthcare Technology R&D Project of the Ministry of Health and Welfare, Korea (HI15C3083).

## CONFLICT OF INTEREST

Authors declare that they have no competing interests.

## Supporting information

Supporting Information

## References

[1] Barnes PJ , Burney PGJ , Silverman EK , et al. Chronic obstructive pulmonary disease. Primer. Nat Rev Dis Primers. 2015;1:15076. 10.1038/nrdp.2015.76 27189863

[2] Cosio MG , Saetta M , Agusti A . Immunologic aspects of chronic obstructive pulmonary disease. N Engl J Med. 2009;360(23):2445‐2454. 10.1056/NEJMra0804752 19494220

[3] Chung KF , Adcock IM . Multifaceted mechanisms in COPD: inflammation, immunity, and tissue repair and destruction. Eur Respir J. 2008;31(6):1334‐1356. 10.1183/09031936.00018908 18515558

[4] Bozinovski S , Hutchinson A , Thompson M , et al. Serum amyloid a is a biomarker of acute exacerbations of chronic obstructive pulmonary disease. Am J Respir Crit Care Med. 2008;177(3):269‐278. 10.1164/rccm.200705-678OC 18006888

[5] Mihailichenko D , Pertseva T . Levels of serum amyloid A (SAA) and C‐reactive protein (CRP) in stable COPD patients and its relationship with disease severity. Eur Respir J. 2017;50(suppl 61): PA3605. 10.1183/1393003.congress-2017.PA3605

[6] Reigstad CS , Lundén GÖ , Felin J , Bäckhed F . Regulation of serum amyloid A3 (SAA3) in mouse colonic epithelium and adipose tissue by the intestinal microbiota. PLoS One. 2009;4(6):e5842. 10.1371/journal.pone.0005842 19513118 PMC2688757

[7] Ye RD , Sun L . Emerging functions of serum amyloid A in inflammation. J Leukoc Biol. 2015;98(6):923‐929. 10.1189/jlb.3VMR0315-080R 26130702 PMC6608020

[8] Davey A , McAuley DF , O'Kane CM . Matrix metalloproteinases in acute lung injury: mediators of injury and drivers of repair. Eur Respir J. 2011;38(4):959‐970. 10.1183/09031936.00032111 21565917

