# Supplementary material for: Serum amyloid A promotes emphysema by triggering the reciprocal activation of neutrophils and ILC3s
Source: Clin Transl Med. 2021 Dec 19;11(12):e637. doi: 10.1002/ctm2.637 (PMC8684768; doi:10.1002/ctm2.637)
Supplement: Supplementary file 1 — Supporting Information [file CTM2-11-e637-s001.docx]

**Supporting Information**

Serum amyloid A promotes emphysema by triggering the reciprocal activation of neutrophils and ILC3s

**Methods and Materials**

**Mice**

Female C57BL/6 mice were purchased from Koatech (Gyeonggi-do, South Korea), and Rag1^-/-^ mice on a C57BL/6 background were purchased from the Jackson Laboratory (ME, USA). All experiments described in this manuscript were approved by the Institutional Animal Care and Use Committee of Seoul National University Hospital (SNUH-IACUC 16-0164-C2A0). Animals were maintained in an Association for the Assessment and Accreditation of Laboratory Animal Care (AAALAC) international-accredited facility (#001169) and were cared for according to the 8^th^ Edition (2010) of the Guide for the Care and Use of Laboratory Animals of the National Resource Council (NRC).

**Murine models of emphysema**

For the lipopolysaccharide (LPS) and porcine pancreatic elastase (PPE)-induced emphysema model in wild-type (WT) or Rag1^-/-^ mice, 10 µg LPS (Merck Millipore, MA, USA) and 0.3 U PPE (Sigma-Aldrich, MO, USA) were suspended in 50 µl PBS and injected intratracheally. For studies on the acute phase of emphysema development, mice were administered with LPS and PPE once (at day 0) and sacrificed 7 days after injection (Fig. S2A). For studies on the chronic phase of emphysema, mice were administered LPS and PPE once a week for 4 weeks and sacrificed 7 days after the final injection (Fig. 1A and S1A). In the SAA-induced emphysema model, 5 µg of recombinant human SAA1 (rhSAA1; PeproTech, NJ, USA) diluted in 50 µl PBS was administrated to the mice intratracheally at day 0. The mice were sacrificed 3 days after the rhSAA1 injection (Fig. 2A).

**Preparation of single-cell suspensions from the lung, liver, and bone marrow**

Lungs were removed from the mice, cut into small pieces, and incubated on a shaker in RPMI-1640 (Welgene, Gyeongsangbuk-do, South Korea) containing 1 mg/ml collagenase type IV (Worthington Biochemical Corporation, NJ, USA) and 0.5 µg/ml DNase I (Sigma-Aldrich) for 1 and a half hours at 37℃. Digested cells were filtered with a 40 µm pore size strainer (SLP Life Science, Gyeonggi-do, South Korea), and red blood cells (RBCs) were lysed with RBC lysis buffer (BioLegend, CA, USA), following the manufacturer’s instructions.

Livers were ground and filtered with mesh. The supernatant was transferred to a new conical tube and centrifuged. The pellet was suspended with 33% of Percoll® Density Gradient Media (GE Healthcare, IL, USA) and centrifuged. Hepatocytes were collected from the supernatant, and non-parenchymal cells (NPC) were collected from the RBC-lysed pellet.

Bone marrow (BM) was obtained from the femurs and tibias of the hind legs of mice. After removing the muscles and tissues surrounding the bones, the bones were flushed with 1 ml RPMI-1640 using a 1 cc syringe. BM cells were filtered with a 40 µm strainer, and RBCs were lysed.

**Flow cytometry**

Single cells from the lung were suspended in PBS and stained using the Zombie Aqua Fixable Viability kit (BioLegend) to exclude dead cells. After washing, the cells were resuspended in FACS buffer (PBS containing 2% bovine calf serum), blocked with anti-CD16/CD32 antibodies (BD Bioscience, NJ, USA), and stained with the following fluorochrome-labeled antibodies for 30 minutes at 4℃: anti-CD3ε (clone 145-2C11), anti-CD11b (M1/70), anti-CD11c (HL3), anti-CD19 (ID3), anti-CD31 (MEC13.3), anti-CD121a (IL-1R1, 35F5), anti-CD49a (Ha31/8), anti-SiglecF (E50-2440), anti-IFN-γ (XMG1.2), and anti-RORγt (Q31-378) (all from BD Bioscience); anti-CD4 (RM4-5), anti-CD45 (30-F11), anti-CD49b (DX5), anti-CD90.2 (30-H12), anti-EpCAM (G8.8), anti-F4/80 (BM8), anti-FcεRIα (MAR-1), anti-I-A^b^ (AF6-120.1), anti-Ki67 (16A8), anti-Ly6G (1A8), anti-NK1.1 (PK136), and anti-IL-17A (TC11-18H10.1) (all from BioLegend); anti-ST2 (RMST2-33), anti-IL-13 (eBio13A) and anti-IL-1β (NJTEN3) (Thermo Fisher Scientific, CA, USA); anti-MMP12 (Bioss Antibodies, MA, USA); and anti-SAA (Cloud-Clone Corp., TX, USA).

To stain human cells from sputum, single-cell suspensions were stained with the following antibodies: anti-CD45 (clone HI30), anti-CD3ε (UCHT1), anti-CD11c (3.9), anti-CD11b (ICRF44), anti-CD14 (HCD14), anti-CD15 (W6D3), anti-CD16 (3G8), anti-CD19 (HIB19), anti-CD24 (ML5), anti-CD49b (P1E6-C5), anti-CD68 (Y1/82A), anti-CD117 (104D2), anti-CD127 (A019D5), anti-FcεRIα (AER-37), anti-NKp44 (P44-8), and anti-IL-17A (BL168) (BioLegend); and anti-ST2L (B4E6) (MD Bioproducts, MN, USA). FACS data were collected on an LSR Fortessa X-20 flow cytometer (BD Biosciences) and analyzed with FlowJo (v10.2) software (FlowJo LLC, OR, USA).

**Quantitative real-time PCR**

Frozen lung and liver tissues were ground in TRIzol Reagent (Thermo Fisher Scientific) with a BioMasher II (Optima, Tokyo, Japan). After homogenization, total RNA was extracted and cDNA was synthesized using the SensiFAST cDNA Synthesis kit (Bioline, London, UK). Quantitative reverse-transcription PCR (RT-qPCR) was performed using the SensiFAST SYBR Lo-ROX kit or the SensiFAST Probe Lo-ROX kit (Bioline). The expression of *Saa1*, *Saa3, Il1β, Il23, Il12A, Il18, Il33, Fpr2, Tlr2,* and *Tlr4* were measured using TaqMan gene expression assays (Thermo Fisher Scientific). The expression of *Mmp1, Mmp2, Mmp8, Mmp9,* and *Mmp12* were measured using PrimeTime qPCR primer assays (Integrated DNA Technologies, IA, USA). Relative expression was determined by normalization to the expression of glyceraldehyde-3-phosphate dehydrogenase (*Gapdh*).

**Histological analysis**

Lung tissues were fixed with 4% of paraformaldehyde (Biosesang, Gyeonggi-do, South Korea) and embedded in paraffin. Paraffin blocks were cut into 4 µm-thick sections and stained with hematoxylin and eosin (H&E).

***In vitro* treatment of immune cells with SAA, cytokines, and neutralizing antibodies**

To stimulate immune cells with SAA1 (Fig. 2F-G, 2M and S4C-D), lineage-negative cells from the lungs were enriched using the Lineage Cell Depletion kit (Miltenyi Biotec, Bergisch Gladbach, Germany) and neutrophils were isolated from BM using the Neutrophil Isolation kit (Miltenyi Biotec). rhSAA1 (1 µg/ml) was added in RPMI-1640 supplemented with 10% FBS and 10 mg/ml gentamicin for 12~72 hours.

To co-culture naïve neutrophils with activated ILC3s (Fig. 4E), neutrophils were isolated from the lungs of untreated mice using the Neutrophil Isolation kit (Miltenyi Biotec) and FACS was used to isolate the ILC3s in the lungs of mice that had been intratracheally treated with LPS/PPE and sacrificed on day 7: thus, Lineage-negative cells were enriched with Lineage Cell Depletion kit (Miltenyi Biotec) and stained with fluorochrome-labeled antibodies against CD45, Lineage (CD3ε, CD19, CD11c, CD11b, F4/80, CD49b, and FcεRIα), CD90.2, ST-2, NK1.1, and CD49a for 30 minutes at 4℃, after which ILC3s were gated as CD45^+^lineage^-^CD90.2^+^ST-2^-^NK1.1^-^CD49a^-^ cells and sorted out with BD AriaIII (BD Biosciences). Thereafter, 1x10^6^ naïve neutrophils (isolated from lungs) and 1x10^5^ activated ILC3s were co-cultured with rmIL-2, rmIL-7, rmIL-1β, and rmIL-23 (20 ng/ml, all from BioLegend) for further stimulating ILC3s ± anti-IL-17A neutralizing antibodies (TC11-18H10.1) (10 μg/ml, BioLegend).

***In vivo* depletion of IL-1β, neutrophils, or ILCs in emphysema model mice**

To block IL-1β or deplete neutrophils in the SAA-induced emphysema model, C57BL/6 mice were injected intraperitoneally with 0.2 mg of *InVivo*Mab anti-IL-1β neutralizing antibodies (B122) (Bioxcell), *InVivo*Mab anti-mouse Ly6G (1A8) (Bioxcell) 1 day before and 1 day after SAA injection (Fig. 3A). To deplete ILCs, Rag1^-/-^ mice were injected intraperitoneally with 0.2 mg of *InVivo*MAb anti-mouse Thy1.2 (CD90.2; 30H12) (Bioxcell) three times, namely, 1 day before LPS and PPE injection and every 2 days thereafter (Fig. 3E).

**Immunofluorescence staining**

1x10^5^ neutrophils were attached to the slide by cytospin and fixed with 4% paraformaldehyde (Biosesang). Then, cells were permeabilized with 0.2% Triton X-100 (Promega, WI, USA) and blocked with 3% bovine serum albumin (BSA) (Thermo Fisher Scientific) in PBS containing 0.1% Tween-20 (Biosesang). Fixed/permeabilized neutrophils were stained using goat anti-human/mouse myeloperoxidase (MPO) polyclonal antibody (R&D systems, MN, USA) and rabbit anti-human/mouse matrix metalloproteinase (MMP12) monoclonal antibody (1A4) (Bioss) at 5 μg/ml for 2 hours at room temperature. Cells were stained with Alexa fluor 488-conjugated anti-goat IgG secondary antibody (Thermo Fisher Scientific) and Alexa fluor 594-conjugated anti-rabbit IgG secondary antibody (Thermo Fisher Scientific) for 2 hours, and covered with ProLong™ Diamond Antifade Mountant with DAPI (Thermo Fisher Scientific) overnight. Slides were analyzed using Nikon A1 Confocal Microscope (Nikon, Tokyo, Japan).

**ELISAs**

SAA levels in the sputum of COPD patients were measured by using the Human Serum Amyloid A1 DuoSet ELISA (R&D Systems), following the manufacturer’s instructions. IL-1β levels in the sputum of the patients were measured with Human IL-1β ELISA Set II (BD Biosciences), following the manufacturer’s instructions.

**Human subjects and sample preparation**

We recruited ~~14 healthy controls and~~ ~~27~~ 50 patients with COPD from the Department of Internal Medicine, Seoul National University Hospital (Seoul, South Korea). All patients were classified into patients with or without emphysema according to results from computed tomography scan. Induced sputum was obtained from the subjects. To eliminate the mucus, induced sputum was treated with 0.1 % dithiothreitol (Sigma-Aldrich), shaken at 37℃ for 20 minutes, filtered through a 70 µm strainer (SLP Life Science) and resuspended in FACS buffer for staining.

**Statistical analysis**

All statistical analyses were performed with GraphPad Prism 7 software. Before statistical analysis, Shapiro–Wilk normality tests were conducted to confirm normality. Two groups were compared by using Mann–Whitney U tests (non-parametric) or unpaired t-tests (parametric). Multiple groups were compared by using Kruskal–Wallis tests followed by Dunn’s post-tests (non-parametric) or one-way analysis of variance (ANOVA) followed by Bonferroni’s post-tests (parametric). Correlations were determined by using Spearman’s correlation coefficient (non-parametric) or the Pearson correlation coefficient (parametric). The data are shown as mean ± standard deviation (SD) or mean ± standard error of the mean (SEM). *P*-values less than 0.5 were considered significant.

**Supplementary tables**

**Table S1. Characteristics of COPD patients**

|  | COPD  without emphysema | COPD  with emphysema | *p* value |
| --- | --- | --- | --- |
| No. of patients | 9 | 41 |  |
| Age (yr) | 67.3±6.4 | 71.0±7.5 | 0.2011 |
| Sx durations (yr) | 5.4±4.7 | 8.5±5.7 | 0.1699 |
| FEV_1_ | 1705±572.9 | 1585±469.7 | 0.5341 |
| FEV_1_(%) | 63.8±13.8 | 60.5±17.1 | 0.6183 |
| FVC | 2755±793.9 | 3280±818.6 | 0.1078 |
| FVC(%) | 76.8±16.2 | 88.3±19.5 | 0.1271 |
| FEV_1_/FVC (%) | 61.7±8.6 | 49.6±13.8 | 0.0233 |
| Atopy, n(%) | 0(0) | 1(2.4) |  |
| Allergic rhinitis, n(%) | 3(33.3) | 4(9.8) |  |
| Smoking history  (never/former/current), n(%) | 4(44.4)/2(22.2)/3(33.3) | 7(17.1)/20(48.8)/14(34.1) |  |

Data are presented as mean ± SDs. FEV_1_, forced expiratory volume in 1 second; FVC, forced vital capacity; N.D, no data

**Supplementary figures and figure legends**

**
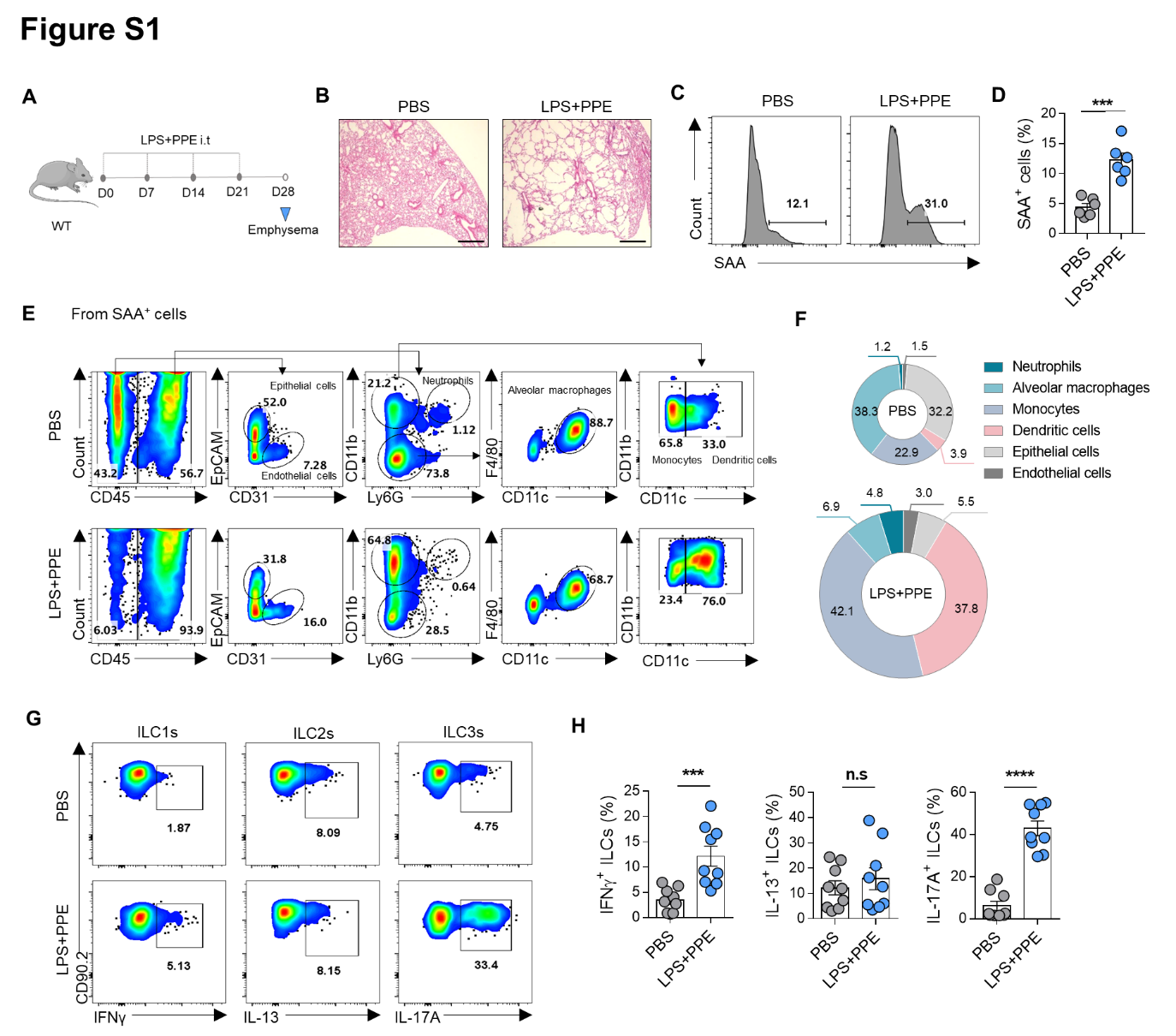
**

**Figure S1. Inflammatory ILC3s increases in emphysema model of WT mice.** (**A**) Schematic representation of the experimental protocol in C57BL/6 mice. To model the chronic phase of emphysema, mice were treated with LPS and PPE at days 0, 7, 14, and 21, and then sacrificed 7 days after the final dose. (**B**) Representative images of lungs from C57BL/6mice. Scale bars, 500 µm. **(C-D)** Flow cytometry analysis of SAA^+^ cells in C57BL/6 mice (C) and the frequency of SAA^+^ cells (D). **(E)** The source of SAA in C57BL/6 mice in emphysema model. **(F)** Pie chart of the percentages of cells secreting SAA in the lungs of C57BL/6 mice. (**G**) Representative flow cytometry dot plots of IFN-γ, IL-13, and IL-17A production by ILCs (CD45^+^Lin^-^CD90.2^+^ cells). (**H)** Comparison of IFN-γ, IL-13, and IL-17A production by ILCs in control mice and mice with emphysema. n.s.; non-significant, ****P* ≤ 0.001, and *****P* ≤ 0.0001 by unpaired t-test. The data are representative of 2–3 independent experiments and are presented as the mean ± SEM.

**
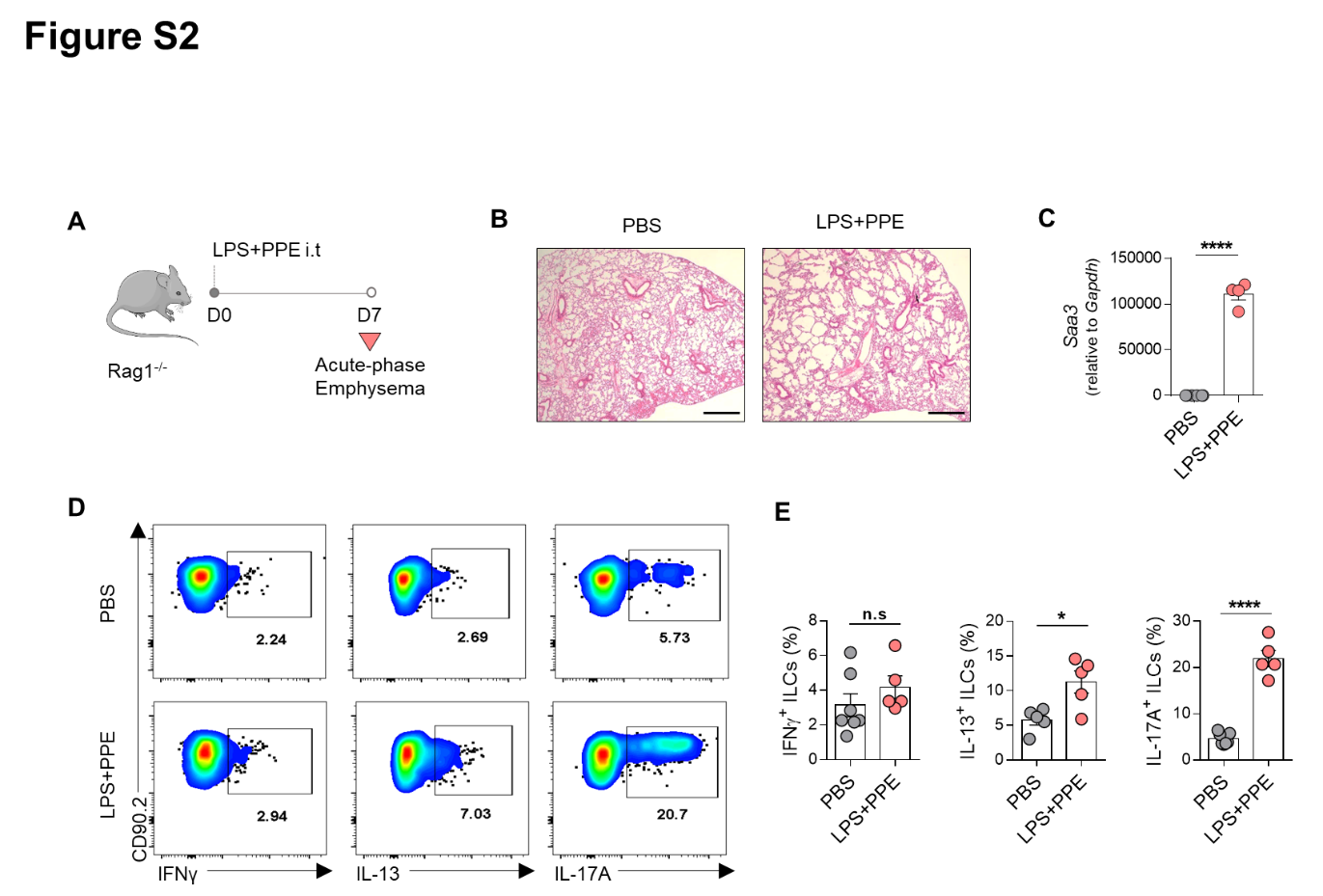
**

**Figure S2. *Saa3* expression and ILC3s increases in acute phase of emphysema model.** (**A**) Schematic representation of the experimental protocol of acute phase of emphysema in *Rag1*^-/-^ mice. To model acute disease, the combination of LPS and PPE was injected intratracheally (i.t.) to C57BL/6 mice at day 0, and the mice were sacrificed 7 days later. (**B**) Representative images of lungs from PBS- or LPS/PPE-treated *Rag1*^-/-^ mice. Scale bars, 500 µm. (**C**) The relative expression of *Saa3* in the lungs of mice with emphysema was quantitatively analyzed. (**D**) Representative flow cytometry dot plots of IFN-γ, IL-13, and IL-17A production by ILCs (CD45^+^Lin^-^CD90.2^+^ cells) in *Rag1*^-/-^ mice. (**E**) Quantification of production of cytokines from ILCs. n.s.; non-significant, **P* ≤ 0.05, and *****P* ≤ 0.0001, by unpaired t-test. The data are representative of 2–3 independent experiments and are presented as the mean ± SEM.

**
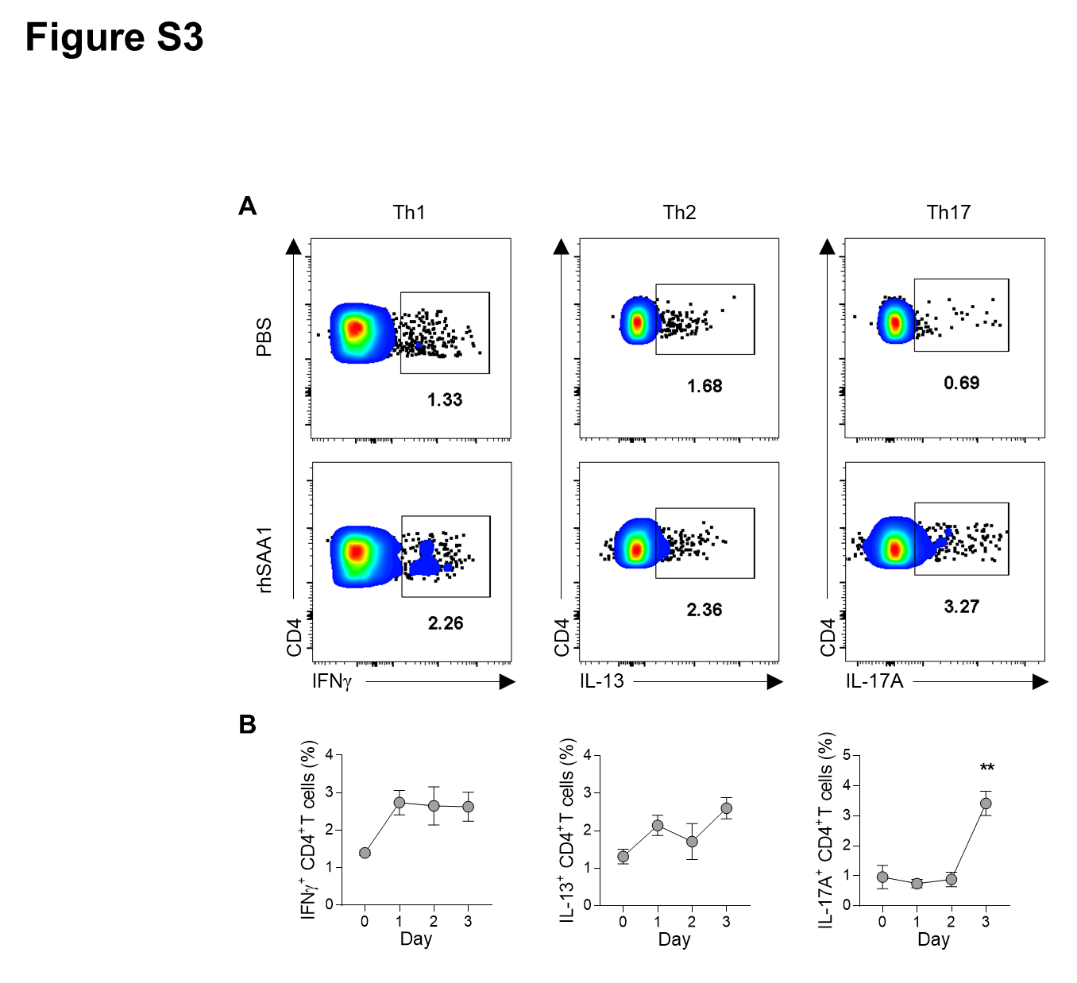
**

**Figure S3. Cytokines expression from CD4^+^ T cells upon SAA administration to lungs**. (**A**) Representative dot plots of IFN-γ, IL-13, and IL-17A production by CD4^+^ T cells (CD45^+^Lineage^+^CD4^+^ cells) 3 days after SAA injection. (**B**) Kinetic analysis of cytokine production by CD4^+^ T cells after SAA injection. ***P* ≤ 0.01, by one-way ANOVA followed by Bonferroni’s post-test. The data are representative of 2–3 independent experiments and are presented as the mean ± SEM.

**
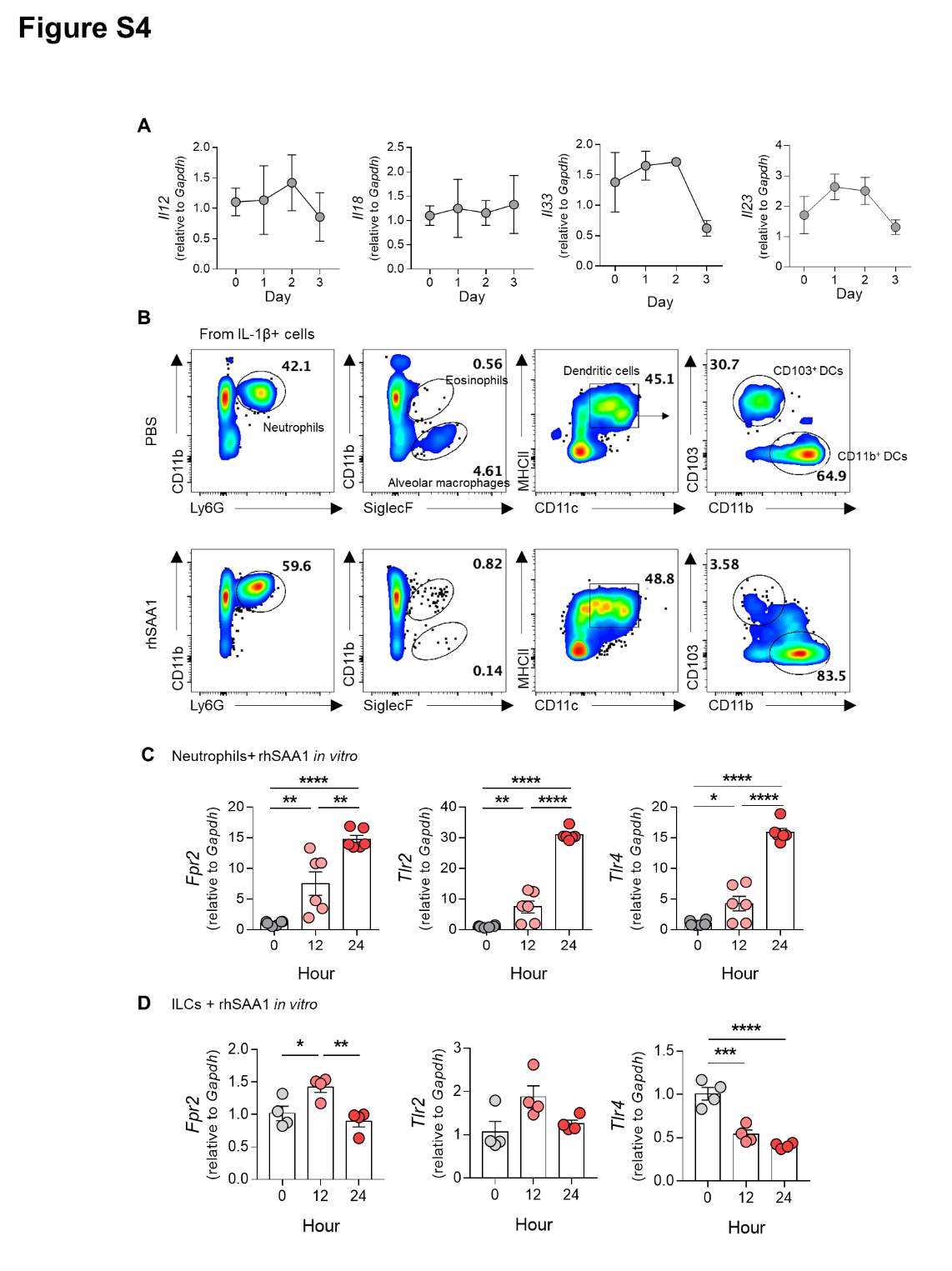
Figure S4. Gene expression of ILC stimulating cytokines in lungs after SAA injection and SAA receptors from ILCs and neutrophils after SAA treatment *in vitro*.** (**A**) Expression of *Il12, Il18, Il33,* and *Il23* in the lungs in the SAA-induced emphysema model. (**B**) Representative dot plot of neutrophils (CD45^+^CD11b^+^Ly6G^+^), eosinophils (CD45^+^CD11b^+^Ly6G^-^SiglecF^+^), alveolar macrophages (CD45^+^CD11b^-^Ly6G^-^SiglecF^+^), CD11b^+^ dendritic cells (CD45^+^Ly6G^-^SiglecF^-^CD11c^+^MHCII^+^CD11b^+^CD103^-^), and CD103^+^ dendritic cells (CD45^+^Ly6G^-^SiglecF^-^CD11c^+^MHCII^+^CD11b^-^CD103^+^) in lungs after SAA treatment. (**C**) Expression of *Fpr2, Tlr2,* and *Tlr4* in bone marrow-derived neutrophils after SAA treatment *in vitro*. (**D**) Expression of *Fpr2, Tlr2,* and *Tlr4* in enriched lung ILCs after SAA treatment *in vitro*. **P* ≤ 0.05, ***P* ≤ 0.01, ****P* ≤ 0.001, and *****P* ≤ 0.0001, by one-way ANOVA followed by Bonferroni’s post-test. The data are representative of 2–3 independent experiments and are presented as the mean ± SEM.

**
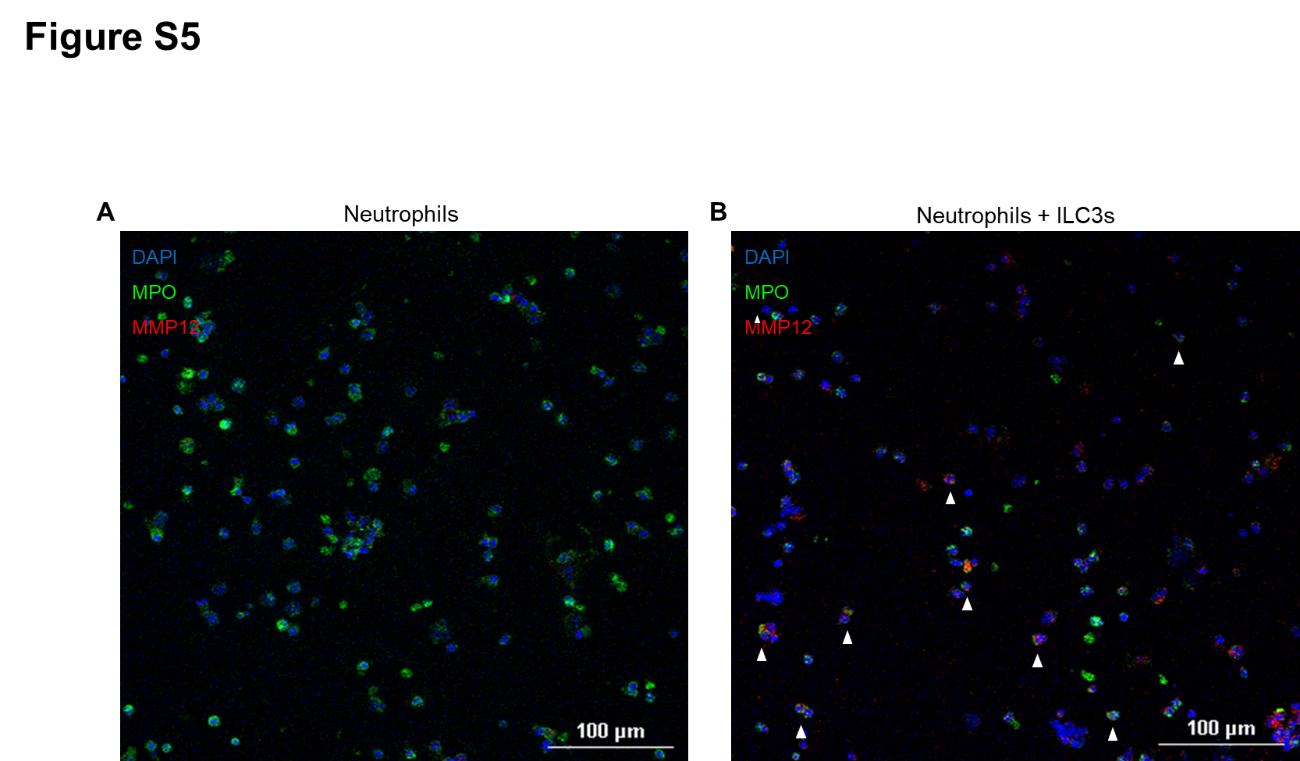
**

**Figure S5. Immunofluorescence image of MMP12 in neutrophils.** (**A**) MMP12 expression from neutrophils in absence of ILC3s. (**B**) MMP12 expression from neutrophils co-cultured with activated ILC3s. Anti-MPO (green), anti-MMP12 (red), and DAPI (blue) were stained for analysis. Scale bars, 100 µm. The data are representative of 2–3 independent experiments.
